# Vitamin D and L-cysteine levels correlate positively with GSH and negatively with insulin resistance levels in the blood of type 2 diabetic patients

**DOI:** 10.1038/ejcn.2014.114

**Published:** 2014-06-25

**Authors:** S K Jain, D Micinski, L Huning, G Kahlon, P F Bass, S N Levine

**Affiliations:** 1 Department of Pediatrics, Louisiana State University Health Sciences Center, Shreveport, LA, USA; 2 Department of Medicine, Louisiana State University Health Sciences Center, Shreveport, LA, USA

## Abstract

**Background/Objectives:**

Vitamin D, L-cysteine (LC) and glutathione (GSH) levels are lower in the blood of diabetic patients. This study examined the hypothesis that the levels of vitamin D and LC correlate with those of GSH in the blood of type 2 diabetic patients (T2D), and that vitamin D and LC upregulate glutamate–cysteine ligase (GCLC), which catalyzes GSH biosynthesis, in cultured monocytes.

**Subjects/Methods:**

Fasting blood was obtained after written informed consent from T2D (*n*=79) and healthy controls (*n*=22). U937 monocytes were pretreated with 1,25 (OH)_2_ vitamin D (0–25 nM) or LC (0–500 μM) for 24 h and then exposed to control or high glucose (25 mM) for 4 h.

**Results:**

Plasma levels of vitamin D, LC, GSH and GCLC protein were significantly lower in T2D versus those in age-matched healthy controls. Multiple linear regression analyses and adjustment for body weight showed a significant positive correlation between plasma levels of vitamin D (*r*=0.26, *P*=0.05) and LC (*r*=0.81, *P*=0.001) and that of GSH, and between LC and vitamin D (*r*=0.27, *P*=0.045) levels. Plasma levels of GSH (*r*=−0.34, *P*=0.01) and LC (*r*=−0.33, *r*=0.01) showed a negative correlation with triglyceride levels. Vitamin D correlated inversely with HbA_1C_ (−0.30, *P*=0.01) and homeostatic model assessment insulin resistance (*r*=−0.31, *P*=0.03), which showed a significant positive correlation with triglycerides (*r*=0.44, *P*=0.001) in T2D. Cell culture studies demonstrate that supplementation with vitamin D and LC significantly increased GCLC expression and GSH formation in control and high-glucose-treated monocytes.

**Conclusions:**

This study suggests a positive relationship between the concentrations of the micronutrients vitamin D and LC and that of GSH. Some of the beneficial effects of vitamin D and LC supplementation may be mediated by an increase in the levels of GSH and a decrease in triglyceride levels in T2D patients.

## Introduction

Reduced glutathione (GSH) is the most prevalent non-protein thiol found in mammalian cells.^1,2^ GSH has a major role in the detoxification of a variety of electrophilic compounds, including peroxides and oxygen radicals catalyzed by glutathione S-transferases, and in the glutathione peroxidase system.^1,2^ In addition, the redox status of GSH has a significant role in signal transduction, gene expression, apoptosis, protein glutathionylation and the maintenance of appropriate protein structure and function.^1,2^ Alteration in GSH levels is associated with a wide variety of pathologies, such as cancer, HIV, lung disease, Parkinson’s disease and diabetes. These studies have led to the free radical theory of human disease and to the advancement of nutritional therapies aimed at improving GSH status under various pathological conditions.^3,4^

GSH is made up of the amino acids glutamine, cysteine and glycine. These amino acids are known to have a direct or indirect role in the maintenance of glucose homeostasis by influencing gene expression and insulin secretion in pancreatic β-cells.^3,4^ High concentrations of glutamate exist in skeletal muscle, coupled with a strong correlation between glutamate and GSH levels, which makes it a major player in the maintenance of glucose metabolism.^5^ Glutamate levels decrease under catabolic conditions in skeletal muscle.^5^ Elevated glucagon levels increase the uptake of glutamine and glycine by the liver, causing the lower blood levels of glutamate, glycine and L-cysteine (LC) seen in diabetes.^3,4,6, 7, 8, 9^ These amino acids are vital for the maintenance of circulating and tissue levels of GSH, which protects against the increased oxidative insults common to diabetes.

Vitamin D blood levels are lower in diabetes and its deficiency is associated with a higher incidence of complications in diabetic patients.^10, 11, 12^ The biochemical pathways influenced by vitamin D in lowering hyperglycemia and its associated complications in diabetes remain to be determined. GSH is formed from LC and L-glutamate by the enzymatic action of glutamate–cysteine ligase (GCLC), and subsequent glycine incorporation catalyzed by glutathione synthase.^1,2^ Whether there is any relationship between circulating levels of vitamin D and LC and those of GSH in diabetic patients is not known. This study reports a positive relationship between plasma levels of LC and vitamin D with those of GSH, which in turn shows a negative relationship with the elevated triglycerides and insulin resistance seen in type 2 diabetic (T2D) patients. In addition, cell culture studies demonstrate that both vitamin D and LC supplementation increases GCLC expression and GSH formation in high-glucose-treated U937 monocytes. Thus, when sufficient nutritional amounts of vitamin D and LC are present in the body, levels of GSH increase, thereby decreasing insulin resistance levels in T2D patients.

## Materials and methods

### Patient enrollment

Informed written consent was obtained from all patients according to the protocol approved by the Louisiana State University Health Sciences Center (LSUHSC) Institutional Review Board. All patients included in this study were healthy adults with type 2 diabetes. All patients who gave written informed consent were invited to return to have blood drawn after fasting overnight. Volunteers for healthy controls were enrolled from a group including siblings of patients or workers at LSUHSC.

### Inclusion/exclusion criteria

Adult T2D patients attending the diabetic clinic at LSUHSC hospital were included in the study. Patients were excluded if they had any history of cardiovascular disease, sickle cell disease, treatment with insulin or metabolic disorders, including uncontrolled hypertension, abnormal parathyroid, hypothyroidism or hyperthyroidism. Patients were excluded if they showed signs of significant hepatic dysfunction, defined as any underlying chronic liver disease or liver function tests >1.5 times the upper limit of normal, or renal dysfunction, defined as a serum creatinine value >1.5 mg/dl. Women with a positive pregnancy test or those nursing infants were also excluded. Subjects who were taking any supplemental vitamins or herbal products were not included in this study.

### Blood collection

Blood was drawn from healthy adults and T2D patients after an overnight fast (8 h). Following blood collection, serum tubes for chemistry profile, EDTA tubes for HbA_1C_ and complete blood counts were promptly delivered to the LSUHSC clinical laboratories. Additional tubes of EDTA blood were brought to the research laboratory. Clear plasma was separated via centrifugation at 3000 r.p.m. (1500 × *g*) for 15 min. All plasma samples were stored at −80 °C for analyses of the biochemical parameters.

### Human pro-monocytic cell line

The U937 monocyte cell line was obtained from American Type Culture Collection (ATCC, Manassas, VA, USA). These cells were maintained at 37 °C in RPMI 1640 medium containing 7 mM glucose, 10% (v/v) heat-inactivated fetal bovine serum, 100 U/ml penicillin, 100 μg/ml streptomycin, 12 mM sodium carbonate, 12 mM HEPES and 2 mM glutamine in a humidified atmosphere containing 5% (v/v) CO_2_. For treatments, cells were washed once in plain RPMI 1640 before being suspended in fresh medium (complete) containing serum and other supplements.^13^

### Treatment with high glucose, LC and vitamin D

Cells (15 ml; 10^6^/ml) were taken in a T-25 cm^2^ cell culture flask and pretreated with LC and 1,25(OH)_2_ vitamin D for 24 h, followed by high glucose (25 mM) exposure for the next 4 h. 1,25(OH)_2_ vitamin D is an active form of vitamin D. In this study control cells were exposed to media with a concentration of 7 mM glucose. In the body, glucose is continuously degraded and re-formed to maintain a 5 mM blood glucose level. However, in cell culture studies, we observed that incubating cells with media with a concentration of 5 mM glucose for 24 h caused a decrease in glucose concentrations to levels <2 mM. In cell culture studies, glucose gets metabolized but not replaced. For this reason, and drawing on our previous experience, we found that using a concentration of 7 mM glucose does not lead to a glucose deficiency after 24- h incubation. In high glucose studies, cells were exposed to a high glucose concentration of 25 mM. In some experiments, cells were instead exposed to 18 mM mannitol (control) as the media contain 7 mM glucose. After treatment, cells were lysed in radioimmunoprecipitation assay buffer for use in western blotting studies.

### GSH, LC, GCLC, vitamin D, HOMA insulin resistance and cell viability assays

GSH and LC levels were determined using high-performance liquid chromatography.^14^ The level of GCLC total protein in the plasma was determined using an ELISA kit (catalog #MBS 704124, MyBiosource, San Diego, CA, USA). The level of GCLC protein was expressed as total protein. Insulin levels in the plasma were determined using the sandwich ELISA method with commercially available kits from Fisher Thermo Scientific Co (Rockford, IL, USA). Plasma levels of 1,25(OH)_2_ vitamin D were determined using an ELISA kit (catalog #VID31-K01, Eagle Biosciences, Nashua, NH, USA). Insulin resistance was calculated from blood glucose and insulin levels using the homeostatic model assessment insulin resistance (HOMA IR) formula.^15^ HOMA IR was calculated using the following formula: (plasma insulin (μU/ml) × glucose (mg%))/405. All appropriate controls and standards as specified by the manufacturer’s kit were used each time. In cell culture studies, the whole cell suspension was processed for each GSH assay, and GSH concentration is expressed per volume of cell suspension. Cell viability was determined using the Alamar Blue reduction bioassay (Alamar Biosciences, Sacramento, CA, USA). This method is based on Alamar Blue dye reduction by live cells.

### Western blotting analyses of cell lysates

At the end of treatment, cells were lysed in radioimmunoprecipitation assay buffer (50 mM Tris pH 8, 150 mM NaCl, 1% NP-40, 0.5% deoxycholic acid and 0.1% SDS) supplemented with protease and phosphatase inhibitors (1 mM PMSF, 5 μg/ml leupeptin, 2 μg/ml aprotinin, 1 mM EDTA, 10 mM NaF and 1 mM NaVO_4_). Lysates were subjected to mild sonication, centrifuged at 15 000 r.p.m. (4 °C, 30 min) and the supernatants collected. Total protein concentrations were determined using the BCA assay (Pierce/Thermo Scientific, Rockford, IL, USA). Equal amounts of protein (20 μg) from each group were loaded onto 10% SDS-polyacrylamide gel after boiling for 5 min with β-mercaptoethanol as a reducing agent. The separated proteins were transferred to a nitrocellulose membrane, blocked with 1% bovine serum albumin in T-PBS (0.25% Tween-20 in phosphate-buffered saline (PBS)), and incubated overnight at 4 °C with the respective primary antibodies using 1:500 dilutions. The next day, membranes were washed with T-PBS (8 min, four cycles) and incubated with secondary antibodies conjugated with horseradish peroxidase in 5% non-fat milk for 30 min at room temperature. The membranes were again washed with T-PBS (8 min, four cycles), treated with chemiluminescence reagents for 2 min and exposed to X-ray films developed through autoradiography. β-Actin antibody was used to assess the loading equality. The antibody for GCLC (73 kDa) was purchased from Abcam (Cambridge, MA, USA). The intensity of each band reported here was measured using ImageJ Software from NIH (Bethesda, MD, USA).

All chemicals were purchased from Sigma Chemical Co (St Louis, MO, USA) unless otherwise mentioned. Data were analyzed statistically using analysis of variance to compare differences between diabetic patients and age-matched healthy subjects. Multiple linear regression analyses were used to determine whether dependent variable can be predicted from a linear combination of independent variables. Body weight was always used as additional independent variable to determine regression and *P-*value. Y-axis parameter as dependent, x-axis and body weight were used as independent variables using Sigma Stat software (San Jose, CA, USA). For cell culture studies, Student’s *t*-test was used to compare the differences between treatments using Sigma plot statistical software (SPSS, San Jose, CA, USA). A *P-*value <0.05 was considered significant.

## Results

### Vitamin D, LC and GSH levels in diabetic patients and control subjects

Table 1 shows the similar distribution of age, F/M ratio, and race in both groups. Body weight and BMI, as well as the blood parameters HOMA IR, triglycerides, and fasting glucose levels, were higher in diabetic patients compared with those of control subjects. Plasma levels of LC, GSH and GCLC are lower in the plasma of T2D patients compared with those of age-matched controls (Table 1). Similarly, plasma levels of vitamin D were lower in T2D compared with those of age-matched control subjects. The vitamin D level was lower whether vitamin D was expressed per nM (volume) of plasma or per cholesterol+TG levels. This difference was examined because vitamin D is a lipid-soluble vitamin similar to vitamin E and it may be more appropriate to express its concentrations after normalization with lipid as has been argued for vitamin E.^16^

### Relationship between vitamin D, LC, GSH, HbA1c and HOMA IR levels in diabetic patients

**Figure 1:**
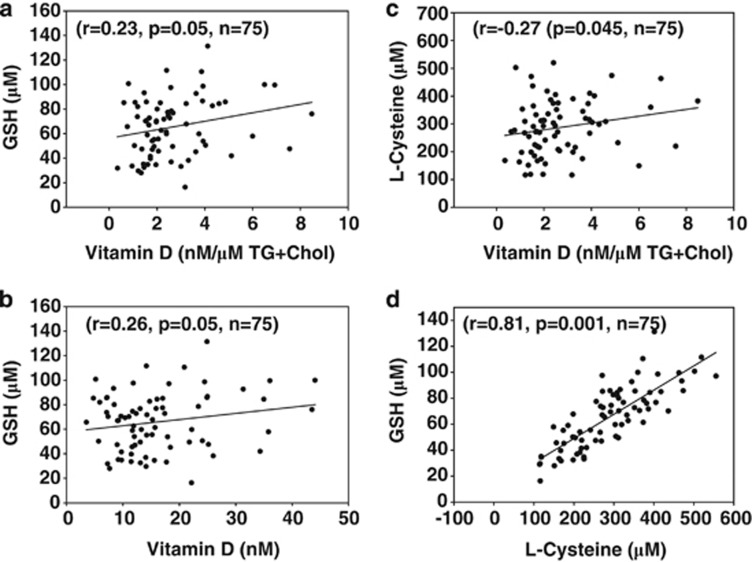
The regression analyses between plasma levels of GSH and L-cysteine (**d**), GSH and vitamin D expressed per lipid (nM/μM triglycerides and cholesterol) (**a**), GSH and vitamin D expressed per nM (**b**) and L-cysteine and vitamin D (**c**) in the plasma of type 2 diabetic patients. Note the significant relationship between GSH and L-cysteine when vitamin D is expressed per lipid or per ml plasma.

Figure 1 shows that blood GSH levels were significantly positively dependent on vitamin D (Figure 1b) and LC (Figure 1d) levels in diabetic patients. This suggests that circulating vitamin D can help boost blood GSH status. Similarly, Figure 1c illustrates that a significant correlation exists between blood levels of LC and those of vitamin D.

**Figure 2:**
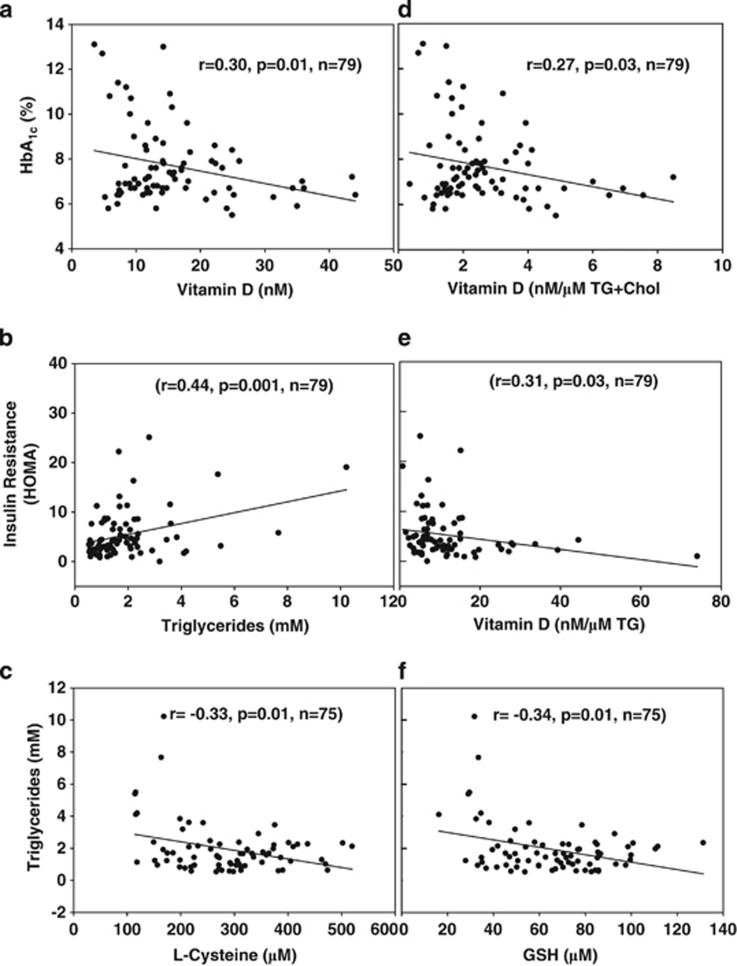
The regression analyses between levels of GSH (**f**) and L-cysteine (**c**) and triglycerides, and vitamin D (**e**) and triglyceride (**b**) and insulin resistance levels in the plasma of type 2 diabetic patients. Note the significant negative relationship between GSH and L-cysteine levels and those of triglycerides; and of HbA_1c_ with that of vitamin D expressed per nM plasma (**a**) or with vitamin D expressed per lipid (nM/μM triglycerides and cholesterol (**d**)) in the plasma of type 2 diabetic patients. Note a significant negative relationship between vitamin D and HOMA IR, and a positive relationship between levels of triglyceride and HOMA IR; and of HbA_1c_ with that of vitamin D status in the blood of type 2 diabetic patients.

Figure 2 shows that there was a statistically significant inverse regression dependence of triglyceride levels with blood levels of GSH (Figure 2f) and LC (Figure 2c), but that the inverse relationship between GSH and cholesterol was not statistically significant (*r*=−0.18, *P*=0.12, data not given). This suggests that blood GSH status has a positive effect on lowering lipid levels. There was a significant inverse regression relation of HOMA IR (Figure 2e) with that of vitamin D, while triglyceride levels were positively correlated with those of HOMA IR (Figure 2b). The relationship between cholesterol levels and those of HOMA IR (not shown here) was also significant (*r*=0.31, *P*=0.01). Figure 2 also shows a significant inverse relationship between HbA1c and vitamin D levels. The regression analyses given here were determined while controlling for body weights using multiple linear regression analyses.

### Effect of vitamin D or LC supplementation on GCLC and GSH in cultured monocytes

**Figure 3:**
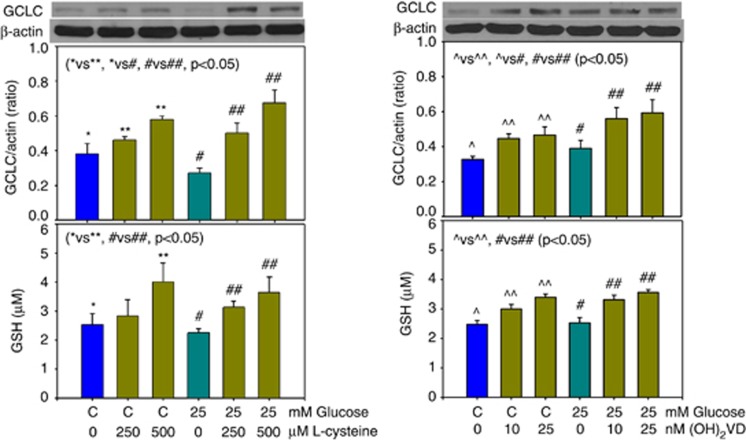
Effect of L-cysteine (left panel) and vitamin D (right panel) supplementation on upregulation of glutamate–cysteine ligase (GCLC) and GSH levels in U937 monocytes cultured with high glucose. Values are mean±s.e. (*n*=3).

GSH is formed from LC by the enzymatic action of GCLC.^17^ To determine whether lower LC and vitamin D levels have any role in lowering GSH levels in diabetic patients, we examined the hypothesis that LC and vitamin D upregulate the GCLC enzyme, which regulates GSH biosynthesis. Figure 3 shows that both LC and vitamin D increase GCLC expression in monocytes treated with control and high glucose. Similarly, there was a modest but significantly higher GSH level in cells supplemented with LC and vitamin D compared with that of respective controls. The effect of vitamin D on GCLC has also been reported in a previous study.^18^ Mannitol treatment had no effect on GCLC or GSH formation (data not given here). There was no change in cell viability in any of the treatments. This suggests that lower levels of LC and vitamin D may have a role in lowering GSH levels in the blood of T2D patients.

## Discussion

This study found lower plasma levels of LC, GSH and GCLC in diabetic patients compared with those in age-matched healthy controls. GCLC catalyzes the first step of the biosynthesis of GSH from LC and glutamate. This suggests that the observed decrease in GSH may be mediated by decreased levels of GCLC in diabetic patients. The lower levels of LC and vitamin D observed in T2D is consistent with those reported in previous studies.^6,7,10, 11, 12^ Multiple linear regression analyses after adjustment for body weight showed a significant correlation between plasma levels of LC and vitamin D and those of GSH levels in diabetic patients. The data also showed a significant inverse correlation between GSH and LC levels and those of triglycerides, which in turn showed a significant positive correlation with HOMA IR levels in T2D patients.

The positive correlation between LC and vitamin D status with GSH levels in diabetic patients is a new finding. To rule out the role of variables such as obesity and parathryroid hormone in diabetic patients, we investigated the direct effect of supplementation of LC and vitamin D on GSH levels in monocytes using cell culture studies. The cell culture studies also demonstrate that the LC and 1,25(OH)_2_ vitamin D caused increased GCLC expression and GSH formation in monocytes. Taken together, the correlation seen in diabetic patients become validated because similar results were obtained with cell culture studies. This suggests that vitamin D and LC status directly and positively influence the GSH levels in the absence of other variables.

Diabetes is associated with elevated blood levels of biomarkers of oxidative stress and vascular inflammation.^19, 20, 21^ Cell culture studies suggest that pro-inflammatory cytokines (such as TNF-α or IL-1β) may cause the release of glutamate and thereby inhibition of LC uptake and decreased GSH synthesis; elevated oxidative stress in various tissues from uncontrolled hyperglycemia may also cause increased utilization of GSH or LC, thereby resulting in the lower GSH levels seen in diabetes.^3, 4, 5,21, 22, 23^ Increased glutamine and glycine uptake by the liver can cause lower blood levels of glutamate, glycine and LC as well as serine—an amino acid important for glycine and LC synthesis, which is also often found to be lower in diabetes.^3,4^ Lower blood levels of vitamin D or LC may be associated with the life style, obesity, vitamin D binding protein and vascular inflammation commonly associated with diabetes.^2,3,21^ This study reports a positive relationship between plasma levels of LC and vitamin D and those of GSH, which in turn shows a negative relationship with the triglycerides and insulin resistance seen in T2D. Thus, sufficient nutritional amounts of vitamin D and LC independently can increase levels of GSH and thereby decrease insulin resistance levels in T2D patients.

Amino acids such as arginine, L-alanine, glutamine and LC are known to stimulate the gene expression of enzymes, such as glutamate dehydrogenase and aminotransferases, as well as insulin secretion in pancreatic β-cells.^4^ Supplementation with cysteine-rich proteins (whey protein and α-lactoalbumin), LC, *N*-acetylcysteine or the cysteinate form of different compounds is beneficial in lowering oxidative stress, insulin resistance and glycemia in diabetic animal and human studies.^24, 25, 26, 27, 28, 29, 30, 31, 32, 33, 34, 35, 36, 37^ Skeletal muscle has an important role in glucose metabolism and contains high concentrations of glutamate and GSH.^5^ Whether supplementation with glutamate, a precursor of GSH, can improve GSH status and glucose metabolism in skeletal muscle needs investigation. Other micronutrients, such as chlorogenic acid, can also increase GSH levels and prevent liver injury.^23^ Intravenous GSH infusion significantly increased the intraerythrocytic GSH/GSSH ratio and total glucose uptake in diabetic patients.^38^ Administration of GSH inhibited the insulin resistance in skeletal muscle caused by lipid infusion in mice^39^ and in Wistar rats.^40^ A recent study reported a positive effect on insulin sensitivity and body composition in older HIV-infected patients as the result of increasing GSH with cysteine supplementation.^41^ Reduced levels of GSH can increase oxidative stress, resulting in the oxidative modification of lipoproteins that can lead to the accumulation of the cholesterol and triglycerides known to induce insulin resistance.^1,2^ Taken together, the results from these studies in the literature and the findings, that a significant negative correlation exists between GSH status and triglyceride levels in T2D patients, indicate that an increase in GSH levels can lower insulin resistance and have a positive effect on glucose metabolism.

The beneficial effects of vitamin D may occur via upregulation of expression of insulin receptors and the glucose transporter, regulation of the calcium pool, modulation of immune and inflammatory cytokine expression (and thereby improvement of insulin action in peripheral tissues) and/or by direct action on pancreatic β-cell function.^10,11,42,43^ This study suggests that some effects of vitamin D are mediated by improvement of the GSH status. Vitamin D is known for its beneficial effects on calcium and other skeletal biomarkers. It is possible that some of the nonskeletal beneficial effects of vitamin D are mediated through its antioxidant action.^44^ Vitamin D is a lipid-soluble vitamin similar to vitamin E. Studies in the literature suggest that the ratio of serum vitamin E to total lipids is the more reliable biochemical index of vitamin E status.^16^ Whether the status of vitamin D is better reflected when expressed per lipid needs further investigation.

In conclusion, this study reports a significant positive relationship between blood levels of LC and vitamin D and those of GSH, and a negative relationship with insulin resistance in T2D patients. New knowledge concerning the nutritional regulation of GSH metabolism may help in the development of effective strategies to improve the health of T2D patients. Future clinical trials are needed to evaluate the potential benefits of supplementation with vitamin D and LC in boosting the status of GSH and lowering hyperglycemia in T2D. If such a trial were to lead to tangible clinical results, it would demonstrate the potential of vitamin D and LC supplementation as an adjuvant therapy in preventing the insulin resistance associated with obesity and diabetes.

## Figures and Tables

**Table 1:** Age, BMI, F/M ratio, fasting glucose levels and insulin resistance, triglyceride, vitamin D, L-cysteine, GSH and GCLC levels in type 2 diabetic patients and controls

	*Type 2 diabetics*	*Controls*
*n*	79	22
Age (years)	49.6±1.03	47.6±1.9
M/F	59/20	16/6
African Americans/Caucasians	54/25	14/8
Body weight (kg)	102.9±3.4	85.1±4.4^a^
BMI	36.6±1.02	29.8±1.3^a^
Blood glucose (mg%)	138.8±6.1	93.2±3.9^a^
HbA_1C_ (%)	7.8±0.2	ND
HOMA IR	5.42±0.5	2.74±0.24^a^
TG (mg%)	171.2±15.4	105.9±12.2^a^
Vitamin D (nmol/l)	15.9±0.96	20.8±1.8^a^
Vitamin D (nM/μM TG+chol)	2.6±0.17	3.2±0.34^a^
L-cysteine (μM)	288.2±11.9	357.6±16.7^a^
GSH (μM)	65.4±2.8	89.4±5.2^a^
GCLC (ng/ml)	0.23±0.03	0.43±0.08^a^

Abbreviations: BMI, body mass index; chol, cholesterol; F, female; GCLC, glycine–cysteine ligase catalytic unit; GSH, glutathione; HOMA IR, homeostatic model assessment insulin resistance; M, male; ND, not determined; TG, triglycerides.

Controls are nondiabetic healthy subjects. HOMA IR was calculated using the formula: (plasma insulin (μU/ml) × glucose (mg%))/405. Values are mean±s.e.

a Values marked were significantly different (*P*<0.05) between the groups.
